# Characterizing veteran and PTSD service dog teams: Exploring potential mechanisms of symptom change and canine predictors of efficacy

**DOI:** 10.1371/journal.pone.0269186

**Published:** 2022-07-27

**Authors:** Clare L. Jensen, Kerri E. Rodriguez, Evan L. MacLean, Ahmad Hakeem Abdul Wahab, Arman Sabbaghi, Marguerite E. O’Haire

**Affiliations:** 1 Center for the Human-Animal Bond, College of Veterinary Medicine, Purdue University, West Lafayette, Indiana, United States of America; 2 Human-Animal Bond in Colorado, School of Social Work, Colorado State University, Fort Collins, Colorado, United States of America; 3 Arizona Canine Cognition Center, School of Anthropology, University of Arizona, Tucson, Arizona, United States of America; 4 Janssen Pharmaceuticals, Titusville, New Jersey, United States of America; 5 Department of Statistics, Purdue University, West Lafayette, Indiana, United States of America; Ryerson University, CANADA

## Abstract

Psychiatric service dogs are an emerging complementary intervention for posttraumatic stress disorder (PTSD). Initial evidence suggests that partnership with a service dog may be related to less PTSD symptom severity. However, it remains unclear how or why this might occur. To address this gap, we conducted a longitudinal investigation of 82 post-9/11 military members or veterans and their PTSD service dogs to (1) evaluate service dog characteristics as potential predictors of efficacy, (2) assess dog and human characteristics as potential predictors of veteran-dog bond, and (3) explore potential mechanisms for mental health outcomes. Aim 1 results demonstrated that most service dog characteristics did not predict veterans’ mental health outcomes, but lower service dog excitability was associated with less PTSD symptom severity at follow-up. Aim 2 results showed that closer dog-veteran relationships were associated with less excitable dog temperament. Aim 3 results indicated that worse mental health at follow-up was associated with greater use of the specifically trained PTSD service dog task to initiate a social greeting (“make a friend”), whereas better mental health was related to less use of dominance-based training methods, lower perceived emotional/logistical costs of service dog partnership, and closer veteran-dog relationships. More frequent use of the trained service dog task to signal when someone approaches from behind (cover/watch back) was associated with greater anxiety, but less PTSD symptom severity. Overall, veterans spent an average of 82% of their time with service dogs (assessed via Bluetooth proximity between dog collar and veteran smartphone), and most frequently asked their service dogs to perform the trained task for calming their anxiety (calm/comfort anxiety). The present study provides subjective and objective metrics of the heterogeneity among veteran-service dog dyads while also suggesting which of the assessed metrics might be potential mechanisms involved in the intervention.

## Introduction

Posttraumatic stress disorder (PTSD) affects a large proportion of military members and veterans with taxing symptoms that can be difficult to manage [1–5]. While several evidence-based treatments and interventions exist for PTSD [6], a growing number of veterans are also incorporating complementary and integrative interventions into their lives to improve daily symptomology and quality of life [7, 8]. One such complementary intervention is animal-assisted intervention in the form of specially trained PTSD service dogs, which have become increasingly popular among military members and veterans in the United States [9]. Paired with an individual person (referred to as the service dog’s *handler*), these dogs are trained to perform tasks for the mitigation of specific PTSD symptoms, such as applying pressure to alleviate anxiety, nudging to interrupt flashbacks, and waking from nightmares [10]. These service dogs are distinct from pet dogs, emotional support dogs, and therapy dogs because their trained tasks are directly related to a disability. This provides service dogs legal protection by the Americans with Disability Act, allowing service dogs public access with their handler [11].

Inspired by salient anecdotes and early qualitative research, preliminary quantitative studies have assessed differences in PTSD symptomology between military members and veterans with and without PTSD service dogs. Several cross-sectional studies have demonstrated that United States military veterans with a PTSD service dog report significantly less symptom severity and better quality of life than those without a service dog [e.g., 12, 13] as well as significantly different stress profiles [14]. In addition to the cross-sectional research, preliminary longitudinal studies have also reported significant improvements in military veterans’ PTSD severity, mental health, and social health after the acquisition of a PTSD service dog [e.g., 15, 16].

Although preliminary research on the psychosocial effects of PTSD service dogs for military veterans is promising, the mechanisms by which PTSD service dogs may bring about these changes remain largely unknown. Theoretical and historical frameworks offer insight into a few potential mechanisms [17]. For example, the social support theory applied to human-animal interactions suggests that service dogs may be a valuable source of non-judgmental companionship for veterans while serving as a catalyst for social engagement with other humans [18]. Companionship received from dogs may also offer similar stress-buffering benefits as one would expect from human social support [e.g., 19]. Further, the biophilia hypothesis suggests that humans are instinctively drawn to other living things in their environment, which may facilitate the effects of service dog interaction for “grounding” a veteran in the present moment and offering a calming presence during distress [20, 21].

Despite a strong theoretical background and encouraging preliminary research, there is insufficient empirical evidence regarding the predictors of efficacy and mechanisms among veteran-PTSD service dog dyads [22]. In the present investigation, predictors of efficacy are defined as independent variables for veterans or service dogs that are significantly associated with the primary intervention outcome (PTSD symptom severity). Mechanisms are defined as the variations in the PTSD service dog intervention itself, such as specific interactions or relationships between veterans and service dogs, through which partnership with service dogs may be related to veteran PTSD severity. Evaluating both predictors of efficacy and mechanisms is critical in the assessment of any intervention and, without rigorous investigation of these components, the intervention cannot be fully evaluated or optimized [23, 24]. Additionally, as research on PTSD service dogs reaches the level of randomized controlled trials (ClinicalTrials.gov numbers NCT03245814, NCT02039843), this information will be vital to the interpretation of current and future outcomes.

As with humans and companion dogs [25], each veteran-service dog dyad is unique and multi-dimensional. Similar to the range of activities and interactions people have with their pet dogs [e.g., 26], the relationships between handlers and their service dogs are not homogenous. For example, differences might exist in personality pairings, activity levels, and signs for affection. In addition to relationship differences, there may also be wide variations in handler and service dog interactions, such as the amount of time they spend together, frequency and type of training the dyad engages in, and veterans’ use of the PTSD-specific tasks their service dogs are trained to do. Understanding these unique features of the intervention could lead to enhancements or modifications to intervention dosage (e.g., time spent together), relationship with the interventionist (e.g., human-animal bond), key elements of the intervention (e.g., use of PTSD-specific service dog tasks), and maintenance of the intervention (e.g., training frequency and methods). Thus, probing heterogeneity in components of the service dog intervention across veteran-service dog dyads has the potential to illuminate mechanisms of therapeutic action, to identify veterans most likely to benefit from these interventions, and to develop best practices for the selection, training and placement of PTSD service dogs.

**Fig 1:**
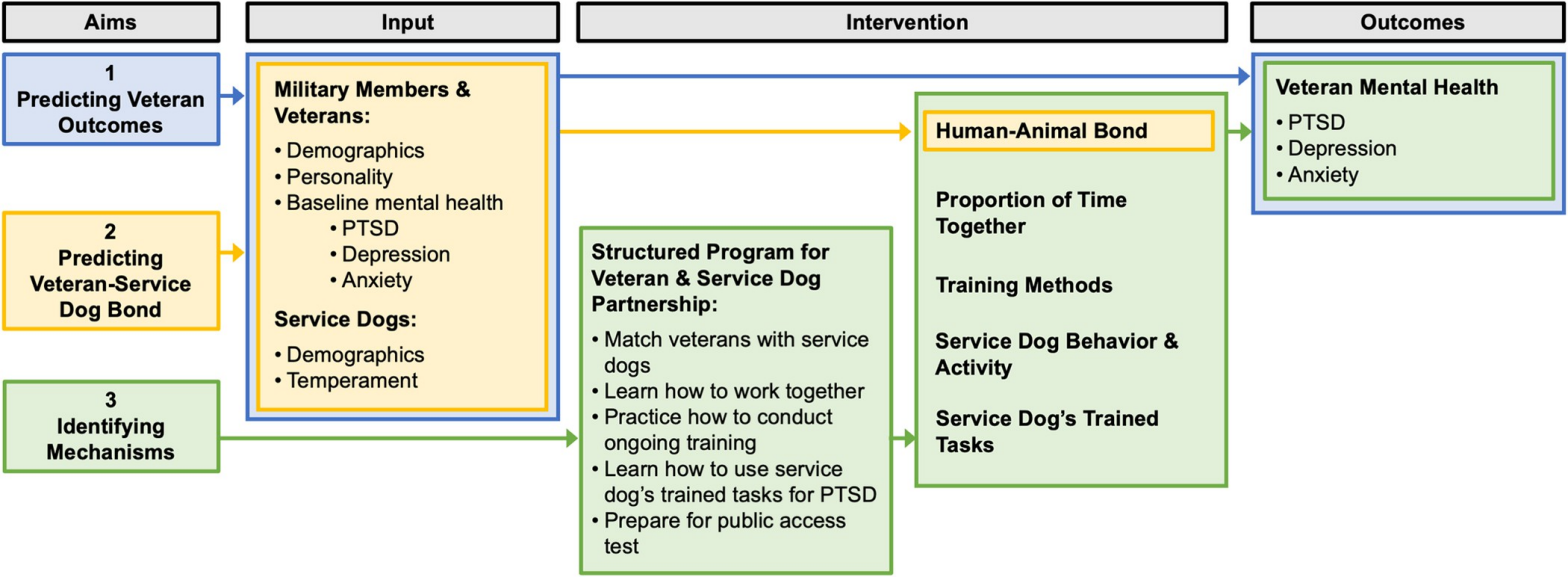
Logic model.

Taken together, there are critical gaps in existing research on the effects that heterogeneity within veteran-dog dyads may have on veteran outcomes. The goal of the present study is to quantify heterogeneity among veteran and PTSD service dog dyads longitudinally to identify canine predictors of veteran outcomes, predictors of the veteran-service dog bond, and potential mechanisms through which the service dog intervention is related to veteran outcomes. We addressed these questions via three specific aims. Aim 1, *predicting veteran outcomes*, was to evaluate the role of service dog baseline characteristics in predicting veteran PTSD and mental health at a three-month follow-up. Aim 2, *predicting the veteran-service dog bond*, was to assess the effects of baseline veteran and service dog characteristics on the subsequent relationship and bond between veterans and service dogs at three-month follow-up. Finally, Aim 3, *identifying mechanisms*, was to evaluate the relationships between human-animal interactions, human-animal bond, and trained service dog task use with veteran PTSD and mental health at three-month follow-up (Fig 1).

## Materials and methods

### Ethics statement

The study protocol was approved by the Purdue University Human Research Protection Program Institutional Review Board (IRB Protocol 1702018766) and by the Purdue Animal Care and Use Committee (PACUC Protocol 1702001541E001). Participants were informed that their data and individual answers would be kept confidential. Voluntary informed consent was obtained verbally by a member of the research team following the review of a written and verbal study description. Consent was also verified electronically at the start of an online survey that was administered as the first screening activity for participation. Participants could choose to discontinue participation or withdraw consent at any time without penalty.

### Participants

The present study consists of 82 military members and veterans assigned to partner with a PTSD service dog from the service dog provider K9s For Warriors (Ponte Vedra Beach, FL, United States), as well as the 82 service dogs with whom they were partnered. K9s For Warriors is an Assistance Dogs International (ADI)-accredited, non-profit organization providing specially trained PTSD service dogs to post-9/11 military members and veterans across the United States at no cost.

Inclusion criteria consisted of both service dog provider-specific criteria as well as study-specific criteria. The provider-specific eligibility criteria for placement with a PTSD service dog from K9s For Warriors included (a) verified PTSD, traumatic brain injury (TBI), or military sexual trauma (MST) diagnosis from a healthcare professional, (b) United States military service after September 11, 2001, (c) honorable discharge or current honorable service, (d) no more than two pet dogs in the home, (e) lack of current substance use, and (f) lack of conviction of any crime against animals. The study-specific eligibility criteria for research participation consisted of acceptance into the K9s For Warriors program (i.e., satisfaction of the provider-specific eligibility criteria) and a PTSD diagnosis by an independent clinician on the Clinician-Administered PTSD Scale (CAPS-5) [27]. There were no exclusions based on demographics (e.g., gender-identity, age), experience with pets, other pet ownership, or prior service dog placements.

All service dogs were screened for trainability, temperament, health, and physical soundness prior to intake by K9s For Warriors. Eligible dogs could be mixed or pure-breed and were acquired from breeders, via direct owner relinquishment, or through animal control, shelters, and rescue organizations. Size requirements included current or anticipated mature weight of at least 50 pounds and height of at least 24 inches. Accepted dogs remained on the K9s For Warriors campus or were temporarily housed by volunteer puppy raisers. Before being placed with a veteran, all dogs were trained for a minimum of 60 hours by K9s For Warriors’ professional dog trainers for basic obedience, appropriate behavior in public, and disability-related tasks.

### Procedure

Participants were recruited from the K9s For Warriors’ database of military members and veterans who had previously applied and been approved to receive a PTSD service dog as part of their routine care. Study invitations were mailed to individuals in the database two months prior to their scheduled placement with a service dog. Invitations included information about study participation and $10 remuneration for time spent reviewing the enclosed information. Mailed invitations were followed by phone calls to answer questions about the study, obtain verbal consent, and schedule screening activities for study eligibility. Screening activities included a brief online survey, through which participants verified informed consent, and the CAPS-5 assessment. The overall response rate was 74% (123/166). Of those who responded, 84% (103/123) enrolled in the study. After study screening, 89% (92/103) of enrolled participants were eligible to participate. Reasons for ineligibility consisted of failure to meet CAPS-5 diagnostic criteria for PTSD (n = 1) and failure to complete screening activities in the timeframe required before service dog placement by K9s For Warriors (n = 10).

**Fig 2:**
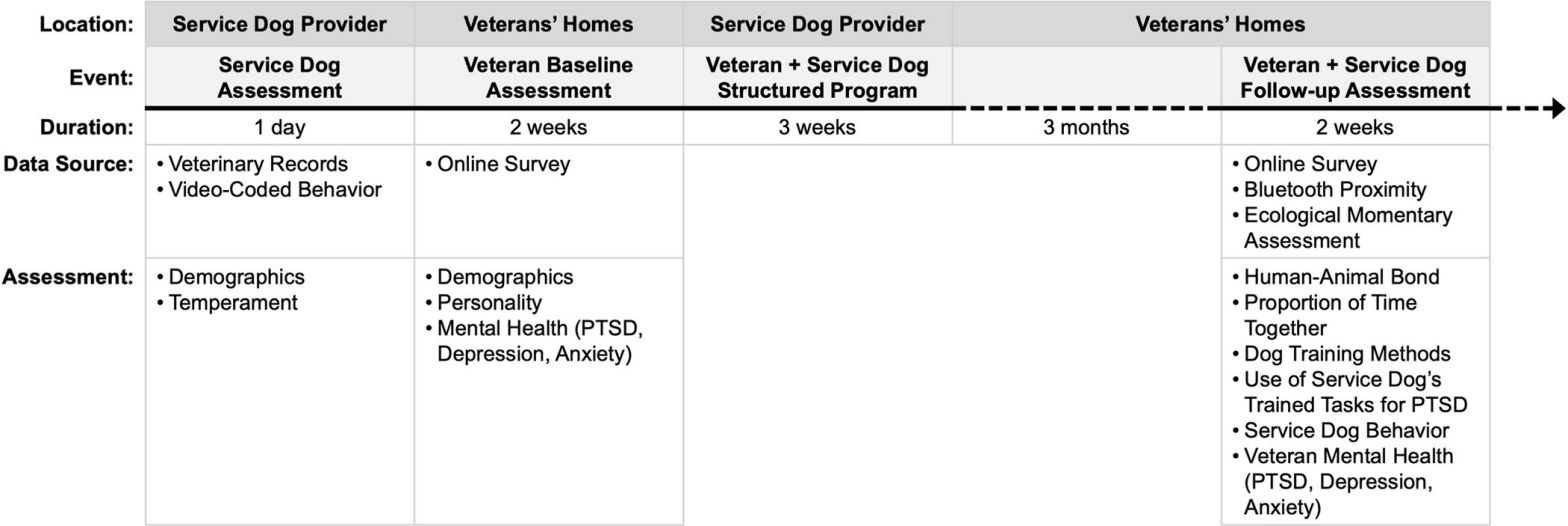
Timeline of study measures.

Study participation consisted of two periods: (1) *baseline* assessment took place in the month preceding PTSD service dog placement, and (2) *follow-up* assessment took place three months later (Fig 2). Both assessment periods occurred in participants’ homes, facilitated by mailed study materials, online surveys, and telephone calls with the research team.

Following the baseline assessment, participants traveled to K9s For Warriors facilities to be placed with their PTSD service dog and complete a three-week structured program. Placements occurred in same-sex groups of 8–12 military members and veterans. K9s For Warriors personnel were blinded to which individuals were participating in the study to reduce any bias. During a three-week structured program, facilitators guided new veteran-dog dyads through a process of getting to know each other, learning the logistics of working together, and practicing trained tasks. Upon completing the three-week program, veterans and service dogs were required to pass a Public Access Test regulated by Assistance Dogs International (ADI) to ensure appropriate behavior of the service dog in public. Following the Public Access Test, military members and veterans returned home with their service dogs.

During the baseline and follow-up study periods, participants completed study activities that were part of a larger, prospective clinical trial (Clinicaltrials.gov ID NCT03245814), including standardized surveys, ecological momentary assessment (EMA), actigraphy monitoring, and salivary cortisol sampling. Data collected at baseline for the present analyses included veteran demographics and mental health (via standardized surveys), and service dog characteristics and temperament (via veterinary records and video-recorded temperament assessments). Data collected at follow-up for the present analyses included veteran-service dog bond (via standardized survey), proportion of time veterans and service dogs were in physical proximity to each other (via a device on the service dogs’ collars with Bluetooth connection to veterans’ phones), relative frequency with which veterans used the PTSD-specific tasks their service dogs were trained for (via EMA), and repeated assessment of veteran mental health.

### Measures

#### Eligibility measures

The *Clinician-Administered PTSD Scale* (*CAPS-5*) [27] was used to determine study eligibility based on positive PTSD diagnosis. CAPS evaluations were conducted via telephone interview by independent and blinded clinicians. Diagnostic criteria of the CAPS include the identification of a specific index trauma (criterion A), at least one symptom of avoidance (criterion B), at least one symptom of intrusive thoughts or feelings (criterion C), at least two symptoms of negatively altered cognition and mood (criterion D), at least two symptoms of heightened arousal and reactivity (criterion E), symptoms lasting more than one month (criterion F), and symptoms causing clinical distress or functional impairment (criterion G).

#### Standardized survey measures

Surveys were administered online via Qualtrics Survey Software and REDCap. Demographics from these surveys included participants’ gender-identity, age, race, ethnicity, marital status, children, and pet dog ownership.

*Ten-Item Personality Inventory (TIPI)*. Veteran personality was quantified at baseline using the TIPI [28]. In this brief measure, two items are used to quantify each of five dimensions of personality (extraversion, agreeableness, conscientiousness, emotional stability, and openness). From the pair of corresponding items, a summary score between 1–7 is produced for each dimension of personality. Higher scores indicate stronger personality traits for a dimension.

*PTSD Checklist for DSM-5 (PCL-5)*. The PCL-5 was used to assess self-reported PTSD symptom severity at baseline and follow-up [29]. The 20-item PCL-5 spans the four PTSD symptom clusters defined in the DSM-5: intrusion (Cluster B, items 1–5), avoidance (Cluster C, items 6–7), negative alterations in cognition and mood (Cluster D, items 8–14), and alterations in arousal and reactivity (Cluster E, items 15–20). Participants were asked to indicate the extent to which they were bothered by each symptom described in an item in the past month. Response options range from zero (not at all) to five (extremely). Responses were summed to create a total PTSD symptom severity score, which could fall between 0–80. Higher scores indicate greater symptom severity. A sum of 33 is the diagnostic threshold for PTSD [30]. Internal reliability on the PCL-5 was high (Cronbach’s alpha = 0.87). Although participants also completed the gold-standard CAPS assessment during eligibility screening, full PCL-5 data was available for a greater number of participants. As the PCL-5 has been validated with CAPS-5 as being psychometrically sound and evidence suggests that PTSD symptom networks are highly robust to measurement methods, PCL-5 was selected over CAPS-5 as our primary outcome measure for PTSD symptom severity [30, 31].

*Patient-Reported Outcomes Measurement Information System (PROMIS)*. Two PROMIS instruments were used to measure mental health in the form of depression (Depression v1.0 adult short form 8a) and anxiety (Anxiety v1.0 adult short form 8a) at baseline and follow-up [32]. These instruments were administered in English via unassisted online self-report. Items in both instruments asked the participant to rate how often they experienced the listed feeling in the prior seven days, with response options on a scale of 1 (never) to 5 (always). Conversion tables published in the PROMIS Adult Profile Scoring Manual were used to convert raw scores to depression and anxiety T-scores, with a population mean of 50 and standard deviation of 10. Higher scores indicate more anxiety or worse depression, respectively. Both short-form instruments have been found to have superior reliability and validity [33] and internal reliability for the present study was high (Cronbach’s alphas depression = 0.94, anxiety = 0.88).

*Monash Dog Owner Relationship Scale (MDORS)*. Three different standardized measures were used to quantify the veteran-service dog bond. These three measures were chosen to each capture a unique aspect of the veteran-service dog relationship. First, the MDORS was used to measure veterans’ perceived relationships with their service dogs at follow-up [34]. A total of 28 items spanning three subscales were used in MDORS: dog-owner interaction (DOI; 9 items), perceived emotional closeness (PEC; 10 items), and perceived costs (PC; 9 items). Costs in this measure are conceptualized as monetary, emotional, and logistical costs associated with dog-ownership (e.g., feeling like the dog is a chore, needing to change plans because of the dog). Participants selected one of five Likert response options for each item, which were summed within subscales to yield total scores between 9–45 (DOI and PC) or 10–50 (PEC). On DOI and PEC subscales, higher scores represent a more positive relationship with one’s dog (i.e., higher level of owner-dog interactions, higher perceived emotional closeness). On the PC subscale, a higher score represents a less positive relationship with one’s dog (i.e., higher perceived costs). The present study had high internal reliability for the subscales of PEC and PC (Cronbach’s alphas = 0.84, 0.88 respectively) and moderate internal reliability for the subscale of DOI (Cronbach’s alpha = 0.61).

*Inclusion of Other in the Self Scale (IOS)*. Second, the IOS was used to measure veterans’ perceived closeness with their service dogs at follow-up. The IOS is a single item scale with seven pictorial response options [35] and has been used in prior measurement of the human-animal bond [e.g., 36]. In the present study, each response option depicted one circle labeled “you” and another circle labeled “service dog” at varying degrees of overlap (from 1 = circles do not overlap to 7 = circles are almost fully overlapping). Participants were asked to select the option most representative of their closeness with their service dog, with a higher score indicating higher perceived closeness. The IOS is a direct and transparent measure asking participants to indicate the closeness of their relationship based entirely on their subjective perception. In contrast, the MDORS PEC subscale produces a calculated score for emotional closeness based on a number of items quantifying specific behaviors and feelings. Although the individual items are still self-report, this method produces a more objective composite score for emotional closeness. The combination therefore allows closeness to be captured in two distinct ways.

*Lexington Attachment to Pets Scale (LAPS)*. Lastly, the LAPS was used to quantify the emotional attachment veterans’ felt to their service dogs at follow-up [37]. In this measure, respondents were asked to indicate how much they agreed with 23 statements about feelings towards their service dog. Response options ranged from 0 (strongly disagree) to 3 (strongly agree). After reverse scoring negatively-worded items, all responses were summed for a total between 0–69, where a higher score indicates stronger feelings of attachment. Internal reliability for LAPS was strong (Cronbach’s alpha = 0.92).

*Training Methods and Behavior*. In addition to the veteran-service dog bond, questionnaires replicated from LaFollette and colleagues [36] were used to assess veterans’ at-home use of various dog training methods and perceptions about their service dogs’ behaviors at follow-up. The two replicated measures had been adapted by LaFollette and colleagues [36] from existing surveys of canine training methods and behavior, including the Canine Behavioral Assessment and Research Questionnaire (C-BARQ^©^) [38–40]. In the first of these measures, veterans were asked to indicate how often they had used each of 17 possible training methods over the prior month on a scale of 0 (never) to 3 (daily). A total training score was calculated as the sum across all 17 training methods, with higher total scores indicating use of a wider variety of training methods with greater overall frequency. Items were subsequently grouped into five training-type categories based on operant conditioning (positive reinforcement, positive punishment, and negative punishment) and interaction (dominance-based and bond-based) training styles. Positive reinforcement was defined as the addition of a rewarding stimulus to promote an increase in the behavior (e.g., praise, using food/treat reward). Positive punishment was defined as the addition of an aversive stimulus to promote a decrease in the behavior (e.g., leash correction, verbal correction such as “no, eh-eh”). Negative punishment was defined as the removal of a rewarding stimulus to promote a decrease in the behavior (e.g., time-out, ignoring an unwanted behavior). Dominance-based training consisted of the belief that humans can influence behavior by asserting dominance over dogs (e.g., staring the dog down until they look away, or forcing dog to roll on their back with “alpha roll”). Bond-based training consisted of the belief that humans can influence behavior by nurturing a close relationship with dogs (e.g., teaching by example with “do as I do”, co-sleeping with dog).

In the second of these replicated measures, veterans were asked to indicate how often their service dog had shown each of 20 behaviors over the prior months on a scale of 0 (never) to 4 (always). Items were grouped into four categories based on what each behavior may indicate. Categories included behaviors indicative of trainability (e.g., coming immediately when called), attachment (e.g., following from room to room when at home), fear/anxiety (e.g., acting cautious or shy around new people), or aggression (e.g., barking or growling at other people or dogs). Items for behaviors indicative of fear/anxiety and aggression were averaged for a single variable to represent overall frequency of problem behaviors.

#### Ecological Momentary Assessment (EMA)

*Use of service dogs’ trained tasks*. Trained task use was measured using Ecological Momentary Assessment (EMA) administered via a smartphone application (RealLife Exp, LifeData). Throughout the 14-day baseline and follow-up study periods, veterans were prompted by the application four times per day (morning, evening, and twice during the daytime) to complete a short survey. Daytime questionnaires were prompted at random intervals with a 30-minute limited response window to capture real-time information with reduced risk of retrospective bias [41]. During the follow-up assessment, within the two daytime questionnaires, participants were asked: “In the last 4 hours, has your service dog done any of the following?” Response options consisted of five tasks for which all K9s For Warriors service dogs were trained: “Interrupt/alert to anxiety”, “Calm/comfort from anxiety”, “Block (create space)”, “Cover (watch back)”, and “Make a friend.” Examples of behaviors associated with these tasks include dogs nudging or placing head in veteran’s lap to “interrupt/alert to anxiety”, laying on top of or leaning against veteran to “calm/comfort from anxiety”, positioning their bodies in front of the veteran to “block (create space)”, positioning their bodies behind the veteran and letting them know if someone is approaching to “cover (watch back)”, and initiating a friendly approach or offering someone their paw to “make a friend [10]. To calculate the relative proportion of use for each of five trained tasks, the sum count for each task was divided by the total number of completed daytime questionnaires for each veteran.

#### Objective canine activity and proximity to human

*Whistle Activity Monitor Devices (Whistle Labs*, *Inc*., *San Francisco*, *CA)*. During the follow-up study period, PTSD service dogs wore unobtrusive collars equipped with Whistle devices, which are tri-axial canine accelerometers with Bluetooth proximity monitoring technology. Accelerometer data was used to calculate an average activity level for each dog. Specifically, Whistle devices provided summarized reports for every dog including minutes of activity and total minutes recorded for each day of data collection. From these summaries, we removed days of zero activity and days during which data was recorded for less than 23 hours, resulting in just over 11 days of activity data per dog (M = 11.3, SD = 2.5). We divided number of minutes active by total minutes recorded to get the proportion of time dogs were active each day, and then calculated the mean of these proportions across all days per dog. Active minutes are reported by the manufacturer to include movement at a higher velocity than what would typically be expected when a dog is changing position or moving casually around the house. As such, minutes of activity included times during which the dog was on a sustained walk with their handler, running, or playing.

*Smartphone application (whistle legacy)*. Proximity monitoring technology was used in tandem with a companion smartphone application (Whistle Legacy) to send Bluetooth pings between the Whistle device and veterans’ phones at 10-minute intervals. If the service dog and veteran’s phone were more than approximately 30 feet apart, the ping would not be recorded in the data. When the service dog and veteran’s phone were less than 30 feet apart, recording of the ping was also conditional on there being few or no substantial barriers between the phone and service dog (e.g., dense walls or structures). Thus, if the veteran’s phone and service dog were less than approximately 30 feet apart with minimal structural barriers between them, the ping was recorded in the data to indicate that they were in proximity at that timestamp.

In subsequent calculations, the number of minutes that elapsed between consecutive ping timestamps was compared to a 15-minute threshold. If the number of elapsed minutes between the consecutive pings was less than 15, veterans and service dogs were considered to be in proximity to each other for the time between those two pings. If it was greater than 15, veterans and service dogs were considered to *not be* in proximity to each other for the time between those two pings. Finally, the sum of all minutes spent in proximity to each other was divided by the sum of all minutes during which the device was collecting data to calculate the mean proportion of time the veteran and service dog were together. To account for extended periods of device removal or malfunction, an additional step removed all time periods in which a veteran and dog were recorded as being apart for more than six hours. This was justified by the fact that, in the corresponding EMA data, veterans consistently reported being with their service dogs during those same time periods. Further, the removal of these time periods did not correspond to any meaningful changes in model results or interpretations.

#### Service dog records

K9s For Warriors maintained records for all service dogs, including veterinary records, dog history forms, transfer of ownership agreement from the original source to K9s For Warriors, training logs, and veteran-signed partnership forms. Two members of the research team manually extracted service dog characteristics from these records, including reported breed, source, sex, and date of birth. Entries were verified by a third member of the research team.

#### Service dog temperament

Per ADI guidelines, all K9s For Warriors service dogs must be temperamentally screened for emotional soundness and working ability prior to placement. Therefore, all service dogs completed a standardized temperament test administered by K9s For Warriors and filmed by the research team, occurring prior or simultaneously to veterans’ baseline assessments. Temperament evaluations consisted of five tests designed to measure the dog’s behavior in different contexts, guided by a single trainer. Tests were given in the same order for all dogs, and were primarily conducted in the same location from the same camera angle (four dogs, or 6% of the sample, received temperament tests in a secondary location). Two to three members of the research team coded videos to quantify five categorical behaviors within the temperament test (Table 1) with adequate reliability (ICC = 0.74). Two members of the research team also coded the number of seconds each dog spent orienting to the trainer using the coding software BORIS [42], with high inter-rater reliability (*r* = 0.92). Orienting to the trainer was operationalized as the dog’s eyes and muzzle being pointed toward the trainer’s head. Based on this definition, videos were first clipped to place a blank frame over segments in which the behavior could not be coded (i.e., any time the dog’s head was not visible, for example, behind the trainer or out of frame). The number of seconds spent orienting to the trainer was divided by total codable seconds, producing a value between 0–1 for the proportion of time the dog spent orienting to trainer according to each rater.

**Table 1:** Ethogram of behaviors scored from service dog temperament tests.

Item label	Item definition	Test description	Scores	Score descriptions
Touch Sensitivity	Response to physical handling	Trainer briefly touches dog’s muzzle, ears, torso, legs and tail	1	Remains still, does not turn head or attend to body where physically manipulated
			2	Dog’s head tracks physical manipulation—paws remain planted, allows physical manipulation.
			3	Dog squirms actively and physically withdraws or attempts to intervene with touching at any point from announcement of task until announcement of next task OR yelps during handling (e.g., crouching, pulling away, or mouthing at hand)
Sound Sensitivity	Response to a sudden loud sound	Trainer drops metal food bowl on ground behind dog	1	Dog does not appear to notice or visibly react to the stimulus. No detectable change in behavior
			2	Dog orients towards sound but shows no major startle or fear
			3	Exhibits transient startle, with immediate recovery. A flinch but no lowering of overall body posture
			4	Lowering of body, bend at the elbow/knee, general fear response
Surface Sensitivity	Reluctance to cross unfamiliar ground surface	Trainer leads dog over wooden trellis on the ground	1	Approaches without visible change in gait (looking or sniffing ok)
			2	Slight hesitation or gait change on approach
			3	Significant pause / change in locomotion before crossing OR attempt to step away, around, or active resistance (pulling backwards)
Food Motivation	Drive for food presented by handler	Trainer offers kibble by hand	1	Follows food persistently—mouth at experimenter’s hand
			2	Distracted, no continual focus on food, has to be reminded of presence of food by trainer
			3	Does not eat food
Approach Excitability	Engagement when approached by an unfamiliar person	Unfamiliar person walks toward the dog and trainer	1	Calm, slow approach. No dramatic change in behavior on approach
			2	Restrained excitement. Speeding up of tail wagging, possible prancing
			3	Eager, excited, unrestrained approach—moves rapidly toward person, jumping or straining at leash
Orienting to the trainer	Proportion of time spent gazing toward trainer’s head		0–1	Number of seconds in which dog’s gaze was directed toward trainer’s head divided by total codable seconds

#### Statistical analyses

Analyses were conducted in two steps. First, elastic net regularization was used for variable selection to minimize overfitting and to identify predictive, parsimonious models [43]. The elastic net methodology effectively combines the regularizers used in LASSO and ridge regression and has the advantage of selecting the active and significant predictor variables from numerous options that may also be correlated with one another. For each outcome variable, we applied elastic net to the full list of potential predictors and covariates for each model to identify those variables that exhibit significant value for predicting the outcome.

In the second step of our analysis, the independent variables selected by elastic net for a particular outcome variable were used as the independent variables in a linear regression model for that outcome. These regression models were fit separately from the elastic net models to obtain unbiased estimates. Regression models utilized the Robust Maximum Likelihood (RML) estimation to yield Huber-White standard errors for the regression coefficients [44]. This method was selected for the purpose of obtaining consistent and unbiased standard errors even in cases of heteroskedasticity (i.e., violation of the constant variance assumption) [45]. Regression coefficients with Huber-White standard errors were subsequently used to assess the magnitude of association between each selected variable and model outcome, controlling for any other variables that were determined by elastic net to be correlated with the outcome variable. Independent variables were standardized prior to analyses. Unstandardized regression coefficients were used to allow interpretation of results in the true scale of each outcome variable. *R*^*2*^ and adjusted *R*^*2*^ values were highly similar for all regression models, and standard (i.e., unadjusted) *R*^*2*^ values were interpreted. The Elastic Net was implemented using a combination of the caret [46] and glmnet [47] packages, while the Huber-White standard errors were estimated using the RCurl [48] package. All analyses were conducted using R version 4.0.5.

The full list of variables considered for addressing Aim 1 (*predicting veteran outcomes*) included service dog demographics, history, and temperament variables, along with covariates for veterans’ gender identity, age, marital status, race and ethnicity, and baseline severity of PTSD, depression, or anxiety (respective to the outcome variable of each individual model: PTSD, depression, or anxiety severity at follow-up). Gender identity, marital status, and race and ethnicity were recoded into three binary variables for analyses. Gender identity was coded as male or not male (i.e., either female or “prefer not to say”) and marital status was coded as single (i.e., either never married, widowed, divorced, or separated) or not single (i.e., either married or living with significant other). Race and ethnicity were combined into one variable whose levels consisted of singularly white (i.e., race is white and ethnicity is not Hispanic/Latinx) or Black, Indigenous, and people of color (BIPOC) and anyone not wishing to disclose (i.e., race is American Indian/Alaska Native, Asian, Native Hawaiian or Other Pacific Islander, Black or African American, more than one race, or prefer not to say, and/or ethnicity is Hispanic/Latinx or prefer not to say). Despite detriments in collapsing distinct identities into over-simplified categories, this strategy was selected to allow for broad consideration of the effects of sample diversity without risking the confidentiality of participants in under-represented demographic groups [49].

Canine temperament scores from video-recorded temperament tests were adjusted prior to analysis to account for potential rater effects. This was carried out by fitting linear mixed models to predict each temperament score as a function of rater identity. Random intercepts from these models were then extracted for each dog, to be used as the rater-adjusted scores in analyses. Based on its score distribution, food motivation was also subsequently recoded to a binary variable prior to any analyses. Dogs who demonstrated the highest level of food motivation were given a score of one and dogs who did not were given a score of zero. When comparing models with food motivation entered as a scale to the same models with food motivation entered as a binary variable, we observed that the model fit and the explained variance were consistently higher in the latter case. Three separate models were run, one each for veterans’ self-reported PTSD symptoms, depression, and anxiety.

To address Aim 2 (*predicting veteran and service dog bond*), service dog demographics, history, and temperament, as well as veteran demographics and personality were considered as independent variables for five human-animal bond-related dependent variables. Whereas veteran demographics were only included in the first aim to control for them as potential confounding variables, they were included in the second aim for their assessment as potential predictors.

Variables considered for addressing Aim 3 (*identifying mechanisms*) consisted of characteristics of the veteran-service dog partnership measured at follow-up. Independent variables for these characteristics included veteran-service dog relationship measures, relative trained task use, daily use of training methods, average dog activity level, proportion of time veterans and service dogs were together, and overall frequency of problem behaviors. Covariates for veteran gender identity, age, marital status, race and ethnicity, and the baseline score of each model’s dependent variable were also considered. Daily positive reinforcement training was excluded because its values were constant (i.e., all participants used positive reinforcement training daily). Since almost all participants used verbal correction daily (n = 61, 95.3%), but only half used any other method of positive punishment (n = 32, 50.0%), the independent variable for punishment-based training methods was recalculated as whether or not participants used any *physical* positive punishment methods daily.

Demographics were available for 82 veteran-dog pairs, but only 60 (73.2%) were included in our analyses due to differential missingness across data streams. Among those with any missing data (n = 28), 28.6% (n = 8) were due to the service dog being returned to the provider during the study period and 39.3% (n = 11) were due to participants voluntarily discontinuing participation in the study entirely. The remaining 32.1% (n = 9) consisted of participants who had voluntarily opted out of some individual study activities, while continuing to participate in others. Compared to dogs remaining with veterans and participants who completed the entire study, there were no significant differences found in any data collected from service dogs that were returned to the provider or participants who discontinued the study. Further, Little’s Missing Completely at Random (MCAR) test yielded a non-significant test statistic, which indicates that missingness and attrition were not related to any observed variables (i.e., the pattern of missingness was MCAR for all variables in analyses).

## Results

### Demographics and descriptors at baseline

Most participating veterans identified as male (78.3%), with a mean age of 36.8 years (± 8.1 years). The majority of the sample was white and not Hispanic or Latinx (75.9%). Most reported to be married or cohabitating with a partner or significant other (57.8%) and most had been honorably discharged from the military (90.2%) as opposed to current honorable service (9.8%). Among participating veterans, the most salient personality trait was conscientiousness (M = 4.6 out of 7, SD = 1.52), whereas the lowest personality traits were emotional stability (M = 2.3, SD = 1.0) and extraversion (M = 2.5, SD = 1.4).

Among participants who completed both baseline and follow-up PCL-5 assessments (n = 78), mean PTSD severity scores were 14.5 points lower after having the service dog for approximately 3 months at follow-up, which is defined as a clinically significant reduction (≥ 10 points) [50]. For those participants who completed baseline and follow-up assessments of depression and anxiety (n = 70), their mean scores for both measures were approximately 6 points lower after having the service dog for approximately 3 months at follow-up, which is greater than a minimally important difference (≥ 3 points) [33]. Additional details on demographics, personality, and background mental health metrics are in Table 2.

**Table 2:** Veteran demographic characteristics, personality, and mental health at baseline.

		*N*	*M* (*SD*) or *n* (%)
**Veteran Demographics**	Age	82	36.79 (8.06)
	Gender Identity	82	
	Male		65 (79.3%)
	Female		17 (20.7%)
	Prefer not to say		0 (0%)
	Race	80	
	American Indian/Alaska Native		0 (0%)
	Asian		0 (0%)
	Black or African American		7 (8.8%)
	Native Hawaiian or Other Pacific Islander		2 (2.5%)
	White		64 (80.0%)
	More than one race		3 (3.8%)
	Prefer not to say		4 (5.0%)
	Ethnicity	82	
	Hispanic or Latinx		15 (18.3%)
	Not Hispanic or Latinx		63 (76.8%)
	Prefer not to say		4 (4.9%)
	Marital Status	82	
	Single (never married)		14 (17.1%)
	Living with significant other		3 (3.7%)
	Married		45 (54.9%)
	Divorced		13 (15.9%)
	Separated		7 (8.5%)
	Widowed		0 (0%)
	Military Employment Status	82	
	Honorably discharged		74 (90.2%)
	Current honorable service		8 (9.8%)
**Veteran Personality**	Extraversion	80	2.49 (1.42)
	Agreeableness	80	3.50 (1.47)
	Conscientiousness	81	4.57 (1.52)
	Emotional Stability	81	2.30 (0.99)
	Openness	81	3.91 (1.21)
**Veteran Mental Health**	PTSD Severity	82	57.01 (11.22)
	Depression	80	64.99 (7.76)
	Anxiety	80	67.28 (6.99)

*Note*. Veteran personality was measured using the Ten-Item Personality Inventory (TIPI) and veteran mental health was measured using the PTSD Checklist for DSM-5 (PCL-5) and PROMIS Depression and Anxiety v1.0 adult short forms, 8a. Veteran personality scores ranged from 1–5 for all five metrics. Veteran mental health scores ranged from 0–80 for PTSD, 38.2–81.3 for depression, and 37.1–83.1 for anxiety.

Service dogs in the present study were primarily male (63.0%) and reported to be mixed-breed (59.3%), with a mean age of 19.7 months on the date they were formally partnered with a veteran (i.e., veteran-service dog team passed public access certification test). Most dogs were sourced from animal control, shelters, and rescue organizations (50.6%) and had been under the care of K9s For Warriors for a mean of 6.4 months at the time of partnership. Among participating dogs, the majority demonstrated the highest level of food motivation (n = 64, 85.3%) and high sensitivity to at least one of sound, surface, or touch (n = 44, 58.7%). Less than half of the dogs were highly excitable by strangers (n = 32, 42.7%). Further information on dog demographics and temperament are in Table 3.

**Table 3:** Service dog demographic characteristics and temperament.

		*N*	*M* (*SD*) or *n* (%)
**Dog Demographics**	Age (months)	81	19.66 (6.48)
	Weight (pounds)	77	60.75 (8.81)
	Sex	81	
	Male		51 (63.0%)
	Female		30 (37.0%)
	Reported Breed	81	
	Labrador Retriever		18 (22.2%)
	Golden Retriever		9 (11.1%)
	German Shepherd		3 (3.7%)
	Poodles and Doodles		3 (3.7%)
	Lab, Golden, or German Shepherd Mix		19 (23.4%)
	Other Mix		29 (35.8%)
**Dog History**	Time with Provider (months)	80	6.43 (4.41)
	Previously Partnered and Returned	81	6 (7.4%)
	Source	81	
	Shelter or rescue		41 (50.6%)
	Owner relinquished directly to provider		5 (6.2%)
	Breeder		24 (29.6%)
	Unknown		11 (13.5%)
**Dog Temperament**	Touch Sensitivity	73	2.45 (0.66)
	Sound Sensitivity	73	2.69 (1.06)
	Surface Sensitivity	73	1.88 (0.63)
	Food Motivation	73	1.10 (0.27)
	Approach Excitability	73	2.40 (0.63)
	Orienting to the handler	73	0.08 (0.05)

*Note*. Possible dog temperament scores range from 1–3 for touch sensitivity, surface sensitivity, food motivation, and approach excitability; 1–4 for sound sensitivity; and 0–1 for orienting to the handler. Breed categories are conditional on the qualifier of *reported* breed, as they were often recorded by shelter or rescue organization staff without information on lineage or genetic testing.

### Veteran-service dog dyadic descriptors

Across all three measures of the human-animal bond, veterans reported high levels of closeness and attachment to their service dogs. Further, on the three MDORS sub-scales, veterans reported frequent dog-owner interactions, strong feelings of emotional closeness, and low perceptions of emotional/logistical cost of service dog partnership.

Of the five training type categories, veterans reported greatest use of positive reinforcement training. Within this category, the most used positive reinforcement methods were physical praise (M = 2.9, SD = 0.40; where a score of 3 indicates daily use and a score of 0 indicates no use) and verbal praise (M = 2.8, SD = 0.82). All participants (100%) reported daily use of at least one positive reinforcement training method and 96.8% of participants reported daily use of two or more positive reinforcement methods. Within the category of positive punishment training, verbal correction was also among the most used training methods (M = 2.9, SD = 0.25), used with the same mean frequency as physical praise. Verbal correction was the only positive punishment used daily for 29 participants (47.5%). Among participants reporting daily use of any physical positive punishment methods (52.5%), n = 25 used leash corrections daily (flat, prong, or both; 41.0%) and n = 7 use physical correction daily (smack, tapping nose; 11.5%).

Using the velocity-based definition of activity, PTSD service dogs in the present sample were active (i.e., on a walk, running, or playing) for a mean of 5.2% of the day. This is equivalent to 74.9 minutes (nearly 1 hour and 15 minutes) per day spent on a sustained walk, running, or playing. Proportion of time in which dogs were active per day ranged from 1.1% to 13.8%. In other words, the least active dog was only active for an average of 15.8 minutes per day, whereas the most active dog was active for an average of 198.7 minutes (3.3 hours) per day.

The objectively measured proportion of time veterans were with their service dogs ranged from 53% to 98% with a mean of 82.3%. The majority of veterans (52%) spent less than 17% of the day apart, equivalent to an average of 4 hours or less per day during which veterans were separated from their service dogs by more than 30 feet or substantial structural barriers (e.g., thick or numerous walls).

The mean response rate to EMA daily check-ins was 86%, for an average of 24 daily check-ins completed per participant over a two-week period. When asked whether they had used a list of tasks in the past four hours, the most frequently reported trained task was to calm/comfort anxiety, reported in 52% of daily check-ins. The next most frequently reported tasks of interrupt/alert to anxiety and “make a friend” were only reported in 18% of daily check-ins each. Means and standard deviations for all dyadic measures are in Table 4.

**Table 4:** Veteran-service dog dyadic descriptors.

		Range	*N*	*M* (*SD*)
**Human-Animal Bond**	Closeness (IOS)	1–7	63	5.68 (1.45)
	Attachment (LAPS)	0–69	63	57.33 (10.34)
	Dog-Owner Interaction (MDORS-DOI)	9–45	63	39.75 (3.74)
	Perceived Emotional Closeness (MDORS-PEC)	10–50	63	43.71 (5.52)
	Perceived Costs (MDORS-PC)	9–45	63	18.02 (7.29)
**Human-Animal Interactions**	Objective Proximity Tracking	0–1	61	0.82 (0.11)
**Veteran Use of Trained Service Dog Tasks**	Interrupt/alert to anxiety	0–1	63	0.18 (0.19)
	Calm/comfort from anxiety	0–1	63	0.52 (0.31)
	Block (create space)	0–1	63	0.16 (0.19)
	Cover (watch back)	0–1	63	0.17 (0.18)
	Make a friend	0–1	63	0.18 (0.22)
**Veteran Use of Dog Training Methods**	Overall Training Frequency	0–3	61	1.47 (0.36)
	Positive Reinforcement	0–3	61	2.15 (0.39)
	Positive Punishment	0–3	61	1.75 (0.50)
	Negative Punishment	0–3	61	0.88 (0.86)
	Bond-Based	0–3	61	0.98 (0.60)
	Dominance-Based	0–3	61	0.86 (0.79)
**Dog Behavior**	Activity	0–1	61	0.05 (0.03)
	Problem Behaviors	0–4	61	0.78 (0.51)

*Note*. IOS = Inclusion of Other in the Self Scale. LAPS = Lexington Attachment to Pets Scale. MDORS = Monash Dog Owner Relationship Scale with subscales. For each category of dog training method, a score of 0 indicates no use of any methods in this category and a score of 3 indicates daily use of every method in this category. Problem behaviors are scored similarly such that 0 indicates no problem behavior and a score of 4 indicates daily problem behavior. Objective proximity tracking, veteran use of trained service dog tasks, and activity are reported as the proportion of time/assessments during which veterans and service dogs were in proximity, veterans were using each trained task, and dogs were active, respectively.

### Predicting veteran outcomes (Aim 1)

From the full list of variables for dog demographics, history, and temperament, as well as covariates for veterans’ gender identity, age, marital status, race and ethnicity, and baseline severity of PTSD, the elastic net procedure selected two variables for the model predicting veteran PTSD at three-months follow-up: veterans’ baseline PTSD severity and dogs’ approach excitability. The subsequent regression model accounted for 34% of variance in PTSD severity at follow-up (*R*^*2*^ = 0.34) and was significantly different from the null model consisting of no independent variables (*F* = 17.42, *p* < .001). Lower veteran PTSD severity at follow-up was significantly associated with less service dog excitability upon approach by an unfamiliar person at baseline (*B* = 3.66, *t* = 2.11, *p* = .038).

The same elastic net procedure applied to the model for veteran depression selected only veterans’ baseline depression as being predictive of depression at three-month follow-up. Next, when applied to the model for veteran anxiety, elastic net selected dogs’ sound sensitivity and response to touch as being predictive of anxiety at three-month follow-up. However, neither of these temperament variables were found to be significantly associated with anxiety in the regression model that followed. Regression coefficients for the independent variables selected by elastic net for PTSD, depression, and anxiety models are in Table 5.

**Table 5:** Regression outcomes for Aim 1 (predicting veteran outcomes), after variable selection by elastic net.

	Predictor	Association with veteran outcomes					
		PTSD		Depression		Anxiety	
	** **	*B*	*SE*	*B*	*SE*	*B*	*SE*
**Dog Temperament**	Touch Sensitivity	-	-	-	-	0.05	0.94
	Sound Sensitivity	-	-	-	-	1.60	0.97
	Approach Excitability	3.66*	1.73	-	-	-	-
**Veteran Control Variables**	Baseline Symptoms	9.51***	1.65	4.15***	1.06	-	-
**Model**	*N*	60		56		56	
	*R* ^*2*^	0.34***		0.19***		0.04	

*Note*. Table excludes variables that were not selected by elastic net for any of the outcomes. Regression coefficients (*B*) and standard errors (*SE*) are unstandardized, Huber-White robust estimates where all variables have been entered into the model as standardized z-scores. Coefficients represent the change in points for the dependent variable associated with one standard deviation increase in the independent variable. Clinically meaningful differences are considered to be 5 points for PTSD and 3 points each for depression and anxiety. Predictors not selected by elastic net included variables for dog demographics (sex, age, weight), dog history (source, time with provider, previous placement), dog temperament (surface sensitivity, food motivation, orienting to the trainer), and veteran control variables (gender identity, age, race/ethnicity, marital status).

- = Predictor was not selected by elastic net for that outcome

***p < .001

**p < .010

*p < .050

### Predicting veteran-service dog bond (Aim 2)

When applied to outcome metrics for interactions, perceived emotional closeness, perceived cost, and attachment, elastic net procedures selected between one to three variables each (see Table 6). However, subsequent regression models showed no significant associations for these four metrics of veteran-service dog bond with any of the variables for dog demographics, history, and temperament, or veteran demographics and personality.

**Table 6:** Regression outcomes for Aim 2 (predicting veteran-service dog bond), after variable selection by elastic net.

	Predictor	Association with veteran-service dog bond									
		Closeness (IOS)		Interactions (MDORS)		Perceived Emotional Closeness (MDORS)		Perceived Costs (MDORS)		Attachment (LAPS)	
		B	SE	B	SE	B	SE	B	SE	B	SE
**Dog Demographics**	Age	-	-	-	-	-	-	-1.36	0.99	-	-
**Dog History**	Previous Placement	-	-	-	-	-	-	-2.51	3.41	-	-
**Dog Temperament**	Food Motivation	-	-	-	-	3.54	2.07	-	-	5.81	3.97
	Approach Excitability	-0.38*	0.18	-	-	-	-	-	-	-	-
**Veteran Baseline Characteristics**	Gender Identity	-	-	-0.69	1.14	-0.84	1.80	-	-	-3.51	3.30
	Marital Status	-	-	-	-	0.65	1.42	-	-	-	-
**Veteran Personality**	Openness	-	-	-	-	-	-	-	-	1.87	1.46
**Model**	N	55		55		55		55		55	
	R^2^	0.07*		0.01		0.07		0.06		0.12	

*Note*. IOS = Inclusion of Other in the Self Scale. LAPS = Lexington Attachment to Pets Scale. MDORS = Monash Dog Owner Relationship Scale. Table excludes variables that were not selected by elastic net for any of the outcomes. Regression coefficients (*B*) and standard errors (*SE*) are unstandardized, Huber-White robust estimates where all variables have been entered into models as standardized z-scores. For categorical independent variables (dog previous placement, food motivation; human gender identity, marital status), coefficients represent the point difference of the entered category (dog had previous placement, high food motivation; human male, single) compared to the reference category (dog had no previous placement, low food motivation; human not male, not single). For continuous independent variables (all remaining), coefficients represent the change in points for the dependent variable associated with one standard deviation increase in the independent variable. Predictors not selected by elastic net for any of the outcomes included variables for dog demographics (sex, weight), dog history (source, time with provider), dog temperament (touch sensitivity, sound sensitivity, surface sensitivity, orienting to the trainer), veteran baseline characteristics (age, race/ethnicity, PTSD, depression, anxiety), and veteran personality (extraversion, agreeableness, conscientiousness, emotional stability).

- = Predictor was not selected by elastic net for that outcome

***p < .001

**p < .010

*p < .050

By contrast, the degree of veteran-dog closeness on the IOS at three-month follow-up was associated with one measure of dog temperament at baseline. Elastic net selected the variable for dogs’ approach excitability and the subsequent regression model accounted for 7% of variance in veteran-dog closeness (*R*^*2*^ = 0.07). This regression model was also significantly different from the null model consisting of no independent variables (*F* = 4.51, *p* = .038). Veterans reported feeling closer to service dogs who had demonstrated less excitability by strangers (*B* = -0.38, *t* = -2.12, *p* = .038).

### Identifying mechanisms (Aim 3)

After elastic net was used to select from variables for human-animal bond, interactions, trained task use, training methods, dog behavior, and veteran demographics, the regression model for veteran PTSD at follow-up explained 49% of the outcome variance (*R*^*2*^ = 0.49). The selected regression model was significantly different from the null model consisting of no independent variables (*F* = 4.09, *p* < .001). Within the model, daily use of dominance-based training was significantly related to worse PTSD, such that veterans who used dominance-based training on a daily basis reported PTSD severity over 11 points higher than veterans who did not (*B* = 11.32, *t* = 2.65, *p* = .011). In contrast, greater use of the trained task to cover (“watch back”) was related to less PTSD severity (*B* = -4.18, *t* = -2.07, *p* = .044). One standard deviation increase in veterans’ use of this task was related to a 4-point decrease in PCL-5 score, where 5 points on the PCL-5 is defined as a reliable difference [50].

The regression model including variables selected by elastic net for veteran depression at follow-up was also statistically significant, explaining 51% of outcome variance (*R*^*2*^ = 0.51, *F* = 5.53, *p* < .001). Within the model, worse depression at follow-up was significantly related to daily use of dominance-based training (*B* = 7.02, *t* = 3.03, *p* = .004) and greater use of the trained task to “make a friend” (*B* = 2.46, *t* = 2.55, *p* = .014).

Finally, the elastic-net selected regression model for veteran anxiety at follow-up explained 40% of the model’s outcome variance (*R*^*2*^ = 0.40), and it was significantly different from the null model (*F* = 4.45, *p* < .001). Less anxiety at follow-up was associated with a greater degree of closeness among veterans and service dogs (*B* = -1.66, *t* = -2.53, *p* = .014). Worse anxiety at follow-up was related to greater perceived costs of service dog partnership (*B* = 3.20, *t* = 3.39, *p* = .001) and greater use of the trained task to cover (“watch back”; *B* = 1.98, *t* = 2.17, *p* = .035). Regression coefficient estimates for independent variables across PTSD, depression, and anxiety models are in Table 7.

**Table 7:** Regression outcomes for Aim 3 (identifying mechanisms), after variable selection by elastic net.

	Predictor	Association with symptoms at three-months follow-up					
		PTSD		Depression		Anxiety	
		B	SE	B	SE	B	SE
**Human-Animal Bond**	Closeness (IOS)	-	-	-	-	-1.66*	0.65
	Attachment (LAPS)	-	-	-1.68	1.25	-	-
	Perceived Costs (MDORS)	2.65	2.08	1.19	1.18	3.20**	0.94
**Human-Animal Interactions**	Objective Proximity Tracking	0.04	1.79	-	-	-	-
**Veteran Use of Trained Service Dog Tasks**	Interrupt/alert to anxiety	-2.47	1.84	-	-	-	-
	Cover (watch back)	-4.18*	2.02	-	-	1.98*	0.91
	Make a friend	-	-	2.46*	0.97	0.58	0.87
**Veteran Use of Dog Training Methods**	Overall Training Frequency	-	-	-1.24	1.24	-1.33	1.13
	Daily Positive Punishment	-4.12	3.87	-	-	-1.66	1.91
	Daily Negative Punishment	-	-	-	-	-1.93	2.39
	Daily Bond-Based	4.27	4.05	-	-	-	-
	Daily Dominance	11.32*	4.27	7.02**	2.31	-	-
**Dog Behavior**	Activity	-1.64	1.89	0.57	1.02	-	-
	Problem Behaviors	-2.14	2.23	-2.31	1.25	-1.48	1.00
**Veteran Control Variables**	Age	-0.26	2.21	-	-	-	-
	Race/Ethnicity	-	-	-5.16*	2.50	-	-
	Baseline Symptoms	8.43***	1.84	4.18***	0.96	-	-
**Model**	N	57		55		55	
	R^2^	0.49***		0.51***		0.40***	

*Note*. IOS = Inclusion of Other in the Self Scale. LAPS = Lexington Attachment to Pets Scale. MDORS = Monash Dog Owner Relationship Scale. Table excludes variables that were not selected by elastic net for any of the outcomes. Regression coefficients (*B*) and standard errors (*SE*) are unstandardized, Huber-White robust estimates where all variables have been entered into models as standardized z-scores. For categorical independent variables (human race/ethnicity; all daily training methods), coefficients represent the point difference of the entered category (human white; training method used daily) compared to the reference category (human BIPOC and anyone not wishing to disclose; training method not used daily). For continuous independent variables (all remaining), coefficients represent the change in points for the dependent variable associated with one standard deviation increase in the independent variable. Clinically meaningful differences are considered to be 5 points for PTSD and 3 points each for depression and anxiety. Predictors not selected by elastic net for any of the outcomes included variables for human-animal bond (MDORS perceived emotional closeness), human-animal interactions (MDORS dog-owner interactions), veterans use of trained service dog tasks (calm/comfort from anxiety, block/create space), and veteran control variables (gender identity, marital status).

- = Predictor was not selected by elastic net for that outcome

***p < .001

**p < .010

*p < .050

## Discussion

We conducted a preliminary investigation of potential predictors of efficacy and mechanisms involved in the partnership between military veterans and service dogs for PTSD. Results described the human, canine, and partnership characteristics of PTSD service dogs for military veterans and members, in addition to identifying several factors associated with partnership effects. Findings complement the growing evidence suggesting that partnership with a PTSD service dog can be an effective complementary intervention for reducing PTSD severity and improving mental health among military members and veterans with PTSD. Specifically, outcomes elucidate how veteran-service dog dyad characteristics can: (1) predict veteran outcomes, (2) predict veteran-service dog bond, and (3) describe the role of specific human-animal interactions as potential mechanisms.

### Predicting veteran outcomes

The first aim of the study was to quantify and evaluate the role of canine baseline characteristics in predicting veteran PTSD severity and mental health at three-months follow-up. Findings indicated that veterans partnered with service dogs who were less excitable when approached by a stranger had less PTSD severity after three months of partnership than veterans partnered with dogs who were more excitable. As a large component of PTSD includes symptoms of hyperarousal and a heightened startle response [51], these data suggest that a calm, less excitable dog may be better suited to help reduce these symptoms than a dog who is more easily excitable. Prior research has also indicated that veterans ascribe many perceived improvements to the calming effects of their service dogs [e.g., 10, 13, 52]. Thus, selecting for dogs with less excitability or promoting these traits in training might increase benefits for the veterans with whom they are partnered.

It is worth noting that all of the dogs included in the analysis were already determined to have low enough excitability for the service dog role. Thus, rather than investigating if excitability relates to whether a dog can become a service dog, our analyses probed whether excitability among pre-selected dogs was related to outcomes of the intervention. Even after dogs with too much excitability were removed from training, and all remaining dogs had undergone long training periods to become less excitable, there were still meaningful differences in excitability among successfully placed service dogs that were associated with their efficacy for reducing PTSD symptom severity. The significant association of dogs’ pre-training excitability with veterans’ follow-up PTSD symptoms suggests potential for optimizing selection and pairing in the future, in that dogs who demonstrate greater excitability (albeit still low enough to succeed as a service dog) might be better suited for a different type of service dog role (e.g., as a hearing assistance dog for someone who is d/Deaf or hard of hearing, where slightly elevated excitability is sometimes considered a favorable trait). Alternatively, this may also suggest that veterans partnered with service dogs who were more excitable before training may need additional support in ongoing management of post-pairing excitability, to ensure that they might access the same benefits as veterans partnered with less excitable dogs.

Whereas veteran PTSD at follow-up could be predicted by service dog temperament, none of the variables for service dog demographics, history, or temperament were found to be predictive of depression or anxiety at follow-up. Consistent with prior research [e.g., 12, 13], military members and veterans in the present sample did report less depression and anxiety after receiving a service dog for PTSD, but these effects were not related to service dog characteristics or temperament. This suggests that demographic variability among service dogs (e.g., mixed vs. pure breeds, animal shelter vs. puppy raiser, differences in sex or weight) does not determine whether or not they are effective as PTSD service dogs. Additionally, this finding suggests that variations in service dog temperament may not be substantial enough to influence veterans’ depression and anxiety. Instead, variations in veterans’ mental health outcomes may be due to other elements of the intervention that are not directly related to canine factors. For example, veterans have reported that simply the responsibility and routine of caring for their service dogs have helped produce improvements in their mental health and well-being beyond symptoms of PTSD [53].

Earlier studies have used canine temperament measures similar to those in the present study to predict the likelihood of success among dogs being trained as guide and assistance dogs [54–56]. For example, dogs demonstrating a calm and nonreactive demeanor have been found to have a higher likelihood of successfully completing service dog training [56]. Due to the small sample size of dogs who were returned to the provider and the largely varied or unknown reasons for each dog’s return, the present study was unable to provide meaningful comparisons for the likelihood of service dogs remaining in the role. However, the finding that canine excitability was associated with PTSD outcomes suggests that measures of temperament have some degree of predictive value for the intervention. Therefore, we expect that future research on the relationship between canine temperament and PTSD service dog success will be valuable.

### Predicting the veteran-service dog bond

The second aim of the study was to quantify and evaluate the role of human and canine baseline characteristics in predicting the bond between veterans and service dogs at three-months follow-up. A multi-modal approach was utilized to quantify the perceived emotional experience of veterans in their partnerships with PTSD service dogs. Across all measures, veterans reported strong positive relationships, attachment, and bond with their service dogs. For example, on the IOS scale of 1–7, 62% of participants (n = 41) selected one of the two highest levels of bond in the form of veteran-service dog closeness. This demonstrates that after only 3 months together, veterans perceive a strong bond with their service dogs.

The bond and quality of the relationship in veteran-service dog partnerships were not associated with most human and canine factors. Yet, the closeness veterans felt with their service dogs after three months of partnership was significantly related to service dog excitability prior to partnership, such that a higher veteran-dog bond was associated with less service dog excitability. This finding is in contrast with findings from a recent study on pet dogs and their owners recruited from the general public in which better relationships were significantly associated with greater owner-reported excitability in pet dogs [57]. However, based on the importance of anxiety and arousal modulation among veterans with PTSD and the different roles of service dogs and pet dogs, it seems reasonable that more excitable service dogs might present challenges to veterans with greater implications than they would have for companion dog owners without PTSD. As the same metric of canine temperament was also associated with PTSD severity at follow-up, this finding suggests that measures of canine excitability prior to partnership may have potential to predict both clinical (i.e., PTSD severity) and emotional (i.e., feelings of closeness) effects for veterans partnered with those service dogs.

The overall PTSD service dog intervention may differ among veterans who have a strong and positive relationship with their service dogs compared to those who feel less bonded [10, 58]. For example, veterans who perceive a positive relationship and strong bond with their service dogs may glean different benefits from the partnership than those reporting a poor relationship. Among people recovering from trauma, research has found that emotional attachment protects against feelings of hopelessness, close bonds contribute to the establishment of a safe and supportive environment, and formation of new affirming interpersonal connections can promote a sense of competency and normalcy [59]. Given this research on the benefits that close relationships among humans may have for trauma recovery, a positive, close relationship with one’s service dog may facilitate similar benefits, independent of symptom changes. However, if the exclusive goal for a military member or veteran with PTSD is to gain a strong emotional bond, then it is possible that comparable benefits may be attained via acquisition of a suitable (e.g., low excitability) pet dog. Thus, it is critical to also examine how the emotional bond with one’s service dog may relate to changes in PTSD symptoms when considered alongside components of the veteran and service dog partnership that are not present in relationships with pet dogs (e.g., public access, specific trained tasks to help with PTSD).

Findings in these areas may also have secondary implications for service dog welfare. A more positive relationship and stronger bond with one’s pet dog has been previously associated with dogs having higher levels of oxytocin, a neuropeptide associated with positive social interactions, mother-offspring attachment, and feelings of relaxation [60, 61]. Additionally, a stronger bond with one’s pet dog has been associated with a higher standard of care evidenced by a greater number of annual veterinary appointments [62]. Based on findings in the present study that service dog temperament may predict bond in the veteran-service dog pair, it will be valuable for future research to directly investigate any connection between human-animal bond and welfare in PTSD service dogs specifically [63, 64].

### Identifying mechanisms

The third aim of the study was to quantify and evaluate the relation of veteran-service dog interactions at three-months follow-up with veteran PTSD and mental health outcomes. Elastic net and regression findings illuminate potential mechanisms involved in the veteran-service dog partnership, through the identification of several factors in the partnership that may be related to PTSD severity and mental health. Our findings suggest that the identified factors (perceived cost of service dog partnership, feelings of closeness with the service dog, daily training methods, and trained task use) may be substantially meaningful and predictive for PTSD, depression, and anxiety.

First, greater feelings of closeness between veterans and service dogs was associated with less anxiety. Based on the social support theory, anxiety may be buffered by support received from close relationships [e.g., 19]. As such, military members and veterans who feel closer to their service dogs may also be receiving more social support from them, thereby promoting larger reductions in anxiety. Greater perceived emotional/logistical costs of the service dog partnership was associated with worse anxiety. Qualitative interviews conducted with veterans who have service dogs for PTSD may provide additional context for this association, as some have described substantial burdens relating to the effort of memorizing specific commands, lack of preparation for taking on a working dog, and new challenges when venturing into the community with a service dog [53]. Thus, the more sacrifices a veteran feels they must make for their service dog partnership, the less they may perceive benefits from that partnership. Alternatively, the less benefit they feel from the partnership, the more of a burden they may find it to care for the service dog. Prior research has found significant associations between greater perceived cost of dog-ownership and higher levels of cortisol in humans, a hormone associated with the stress response system [60]. Additionally, greater perceived emotional/logistical costs of pet dog-ownership has been found to be significantly correlated with greater long-term stress in dogs themselves [57]; thus, both members of the dyad may experience psychological detriments when perceived costs are high.

Veterans reported use of assorted training methods, with all participants reporting use of at least one training method daily. All participants used positive reinforcement every day, alone or in various combinations with positive punishment, negative punishment, bond-based, and dominance-based training methods. Daily use of different training types among veterans may provide additional context for the emotional valence of these regularly occurring veteran and service dog interactions. Indeed, daily use of dominance-based training was related to worse PTSD symptom severity and depression. Considering evidence that punitive training methods create stress and negative welfare in dogs [65], techniques centered around assertion of dominance may compromise the formation of a healthy dyadic relationship. However, based on the exploratory nature of the present study, additional research with greater temporal specificity would be necessary to expand on these training effects and uncover potential confounding factors (e.g., if certain behaviors elicit greater use of specific training methods, or if greater PTSD severity increases the likelihood of using dominance-based methods). These questions are particularly salient as some programs implement the intervention with greater emphasis on the veterans themselves training pets or untrained dogs to become service dogs [e.g., 66, 67].

An objective measure of physical proximity elucidates for the first time the amount of time veterans are actually spending with their service dogs in their day-to-day lives. Findings from this measure indicated that, on average, veterans spend the vast majority of time (82%) with their service dogs. Although all veterans spent more time with their service dogs than they did without them, their time together ranged from 12 hours and 43 minutes to 23 hours and 31 minutes together on an average a day. The lack of a significant relationship between time together and mental health outcomes suggests that service dogs’ effects for veterans may not be dependent on them constantly being together. However, as the lowest proportion of time together in the present sample was still over 50%, this may not be the case if veterans spend less than 50% of time with their service dogs. Although the observed proportion of time spent with service dogs was lower than expected for several participants, there are several scenarios that might explain these low proportions for some veteran-service dog dyads. For example, it is possible that some veterans had jobs in which their service dogs could not accompany them. Further, it is important to note that proximity measurement was based on Bluetooth signaling between the device on the dog’s collar and the veteran’s smartphone. As such, reported proximities may have been inaccurate at times if the collar was removed or the veteran was away from their phone. Although participants were instructed to keep their phones with them throughout the study period, this may not have always been possible during certain activities (e.g., swimming) or for participants who may have been required to leave their phones in lockers or keep them powered off during work hours.

In contrast to relying on participants’ memories for how often they might have used a specific task over the past two weeks, EMA captures this information in real-time with greater ecological validity [68]. This novel and objective metric thereby provides greater rigor for collecting quantified information on the individual processes and experiences within a veteran and service dog partnership. Among EMA quantifications of veterans’ trained task use, the finding that the most-used task is to calm/comfort from anxiety replicates prior findings with this population [10] and supports prior research and qualitative reports that stress and anxiety-modulation is the most helpful or important factor in the veteran-service dog partnership [e.g., 10, 13, 52]. Not only are service dogs’ trained tasks used frequently, several tasks were also significantly related to outcomes in PTSD, depression, and anxiety. Greater use of the trained task to “make a friend” was associated with greater depression, suggesting that veterans with worse depression may be more likely to need or want to use this task. Further, greater use of the trained task to cover (“watch back”) was associated with less PTSD severity, but worse anxiety. This might suggest that worse anxiety may prompt veterans to use this task more frequently, but that this more frequent use may then also bring about improvements in other symptoms of PTSD. However, it is important to keep in mind the observational nature of this study. Without sufficient data to determine direction of causality in results that were somewhat surprising, these interpretations can only be considered as speculations. Future research with additional assessment periods and greater temporal specificity is essential to providing context for these findings.

The preliminary identification of specific trained tasks, training methods, and bond metrics related to veteran outcomes exemplifies potential mechanistic pathways through which service dogs might lead to symptom changes. Prior to the present study, necessary empirical evidence on potential mechanisms was minimal. As an understanding of mechanisms is critical to advancements in human-animal interaction theory and animal-assisted intervention optimization (including in the form of service dogs for PTSD), these preliminary findings demonstrate an important opportunity to move the field forward through the provision of a foundation on which to build future research. Continued examination of intervention refinement focused on these tasks, training, and relationship development, along with other potentially promising mechanisms, could lead to targeted therapeutic enhancements. Future research will be necessary to identify causality and directionality beyond these initial exploratory findings.

### Limitations

Despite novel findings in this exploratory investigation, it is not without limitations. First, measurement limitations could have affected survey instruments and veteran-dog proximity via Bluetooth. Although self-report may be highly valuable for describing one’s own lived thoughts and experiences (i.e., mental health outcomes, personality), other outcomes we measured (i.e., dog problem behaviors, training frequency) may be subject to potential social desirability, expectancy, and/or recall biases. Indeed, self-report has been found to be less accurate for rating dog behavior compared to objective behavioral coding [69]. Both subjective and objective canine behavior ratings were incorporated into the study. Veterans’ ratings of human-animal bond are also subject to positive reporting bias, as many have undergone extensive waiting times to be partnered with their service dogs and there may be pressure to make the partnership work. Additionally, the Bluetooth method used for proximity measurement may have also been subject to inaccuracy based on participants’ smartphone habits or environmental conditions impacting Bluetooth signals.

Second, sampling limitations of a small sample size from a large and varied population may compromise generalizability of the present findings. Due to veteran-service dog pairs being recruited from a single service dog provider, results may not generalize to other providers. Self-selection bias limits the findings to only those who seek to apply for a service dog and who are approved through the specific provider. As many factors may dictate whether or not a person is amenable to being placed with a service dog, it seems unlikely that findings would generalize to veterans who do not wish to receive a service dog, or even to those who are not opposed but have not actively sought one out. Similarly, differences among those who were willing and able to participate in this research may have been an additional source of bias. Further, limited racial and ethnic diversity in the present sample is not representative of the post-9/11 military member and veteran population. For example, based on census survey data collected from this population in 2018, approximately 15% of post-9/11 veterans are Black [70], whereas only approximately 9% of the present sample identified as Black or African American. It is possible this might be an effect of differential desire to work with a service dog, as some have proposed racial and ethnic variance in social stigma around and feelings toward dog-ownership [71, 72]. However, this may also be a reflection of racial and ethnic disparities found in PTSD treatment initiation [73] and in clinician referrals to additional PTSD interventions [74]. Research has indeed demonstrated that veterans who are Black, indigenous, and people of color may be less likely to receive certain types of PTSD interventions, independent from any differences in need, access, and beliefs about treatment [74]. In any case, the lack of diversity in the present sample reinforces a critical need for future research regarding diversity, equity, and inclusion in animal-assisted interventions, as well as the critical need to assess cultural competency in PTSD treatment [73].

Finally, findings must be considered within the caveats of this being a preliminary and exploratory study from which we cannot determine the causality of effects. By nature of the timeframe in which veteran and service dog interactions were quantified with PTSD and mental health outcomes, we are unable to deduce the directionality of effects from human-animal interactions (Aim 3). For example, we are unable to determine whether greater feelings of veteran-service dog closeness led to reduced anxiety, less anxiety led to increased feelings of veteran-service dog closeness, or closeness and anxiety both covaried with a different causal variable that was unmeasured. Thus, our findings suggest potential mechanistic pathways that are promising for further research. It is also possible that the nature of the service dog partnership may be meaningfully beneficial in ways that are not yet appropriately measured.

## Conclusion

Building on existing evidence of service dog partnership being significantly associated with less PTSD severity and better mental health for some veterans, the results of our study begin to illuminate possible means by which reduced PTSD and improved mental health may occur from the PTSD service dog partnership. Findings from this exploratory study set the stage for future research to rigorously test the role of canine characteristics and human-animal interactions preliminarily identified to be important in producing clinical effects.

## References

[1] Fulton JJ, Calhoun PS, Wagner HR, Schry AR, Hair LP, Feeling N, et al. The prevalence of posttraumatic stress disorder in Operation Enduring Freedom/Operation Iraqi Freedom (OEF/OIF) Veterans: A meta-analysis. J Anxiety Disord. 2015 Apr;31:98–107. doi: 10.1016/j.janxdis.2015.02.003 25768399

[2] Galovski T, Lyons JA. Psychological sequelae of combat violence: A review of the impact of PTSD on the veteran’s family and possible interventions. Aggress Violent Behav. 2004 Aug;9(5):477–501.

[3] Hoge CW, Grossman SH, Auchterlonie JL, Riviere LA, Milliken CS, Wilk JE. PTSD Treatment for Soldiers After Combat Deployment: Low Utilization of Mental Health Care and Reasons for Dropout. Psychiatr Serv. 2014 Aug;65(8):997–1004. doi: 10.1176/appi.ps.201300307 24788253

[4] Pacella ML, Hruska B, Delahanty DL. The physical health consequences of PTSD and PTSD symptoms: A meta-analytic review. J Anxiety Disord. 2013 Jan;27(1):33–46. doi: 10.1016/j.janxdis.2012.08.004 23247200

[5] Tanielian TL, Jaycox L. Invisible wounds of war: psychological and cognitive injuries, their consequences, and services to assist recovery. Santa Monica, CA: RAND; 2008. 453 p.

[6] VA/DOD Clinical Practice Guideline for the Management of Posttraumatic Stress Disorder and Acute Stress Disorder. 2017;200. 10.1176/appi.focus.16408 PMC6996084 32021581

[7] Libby DJ, Pilver CE, Desai R. Complementary and Alternative Medicine in VA Specialized PTSD Treatment Programs. Psychiatr Serv. 2012 Nov 1;63(11):1134–6. doi: 10.1176/appi.ps.201100456 23117511

[8] Wynn GH. Complementary and Alternative Medicine Approaches in the Treatment of PTSD. Curr Psychiatry Rep. 2015 Aug;17(8):62. doi: 10.1007/s11920-015-0600-2 26073362

[9] Walther S, Yamamoto M, Thigpen AP, Garcia A, Willits NH, Hart LA. Assistance Dogs: Historic Patterns and Roles of Dogs Placed by ADI or IGDF Accredited Facilities and by Non-Accredited U.S. Facilities. Front Vet Sci [Internet]. 2017 [cited 2020 Sep 24];4. Available from: doi: 10.3389/fvets.2017.00001 28154816 PMC5243836

[10] Rodriguez KE, LaFollette MR, Hediger K, Ogata N, O’Haire ME. Defining the PTSD Service Dog Intervention: Perceived Importance, Usage, and Symptom Specificity of Psychiatric Service Dogs for Military Veterans. Front Psychol [Internet]. 2020 [cited 2021 Apr 3];11. Available from: https://www.frontiersin.org/articles/10.3389/fpsyg.2020.01638/full 10.3389/fpsyg.2020.01638 PMC7396623 32849004

[11] ADA 2010 Revised Requirements: Service Animals [Internet]. [cited 2021 Jul 22]. Available from: https://www.ada.gov/service_animals_2010.htm

[12] O’Haire ME, Rodriguez KE. Preliminary efficacy of service dogs as a complementary treatment for posttraumatic stress disorder in military members and veterans. J Consult Clin Psychol. 2018 Feb;86(2):179–88. doi: 10.1037/ccp0000267 29369663 PMC5788288

[13] Yarborough BJH, Owen-Smith AA, Stumbo SP, Yarborough MT, Perrin NA, Green CA. An Observational Study of Service Dogs for Veterans With Posttraumatic Stress Disorder. Psychiatr Serv. 2017 Jul;68(7):730–4. doi: 10.1176/appi.ps.201500383 28292227

[14] Rodriguez KE, Bryce CI, Granger DA, O’Haire ME. The effect of a service dog on salivary cortisol awakening response in a military population with posttraumatic stress disorder (PTSD). Psychoneuroendocrinology. 2018 Dec;98:202–10. doi: 10.1016/j.psyneuen.2018.04.026 29907299 PMC8454180

[15] Bergen-Cico D, Smith Y, Wolford K, Gooley C, Hannon K, Woodruff R, et al. Dog Ownership and Training Reduces Post-Traumatic Stress Symptoms and Increases Self-Compassion Among Veterans: Results of a Longitudinal Control Study. J Altern Complement Med. 2018 Dec;24(12):1166–75. doi: 10.1089/acm.2018.0179 30256652

[16] Kloep ML, Hunter RH, Kertz SJ. Examining the Effects of a Novel Training Program and Use of Psychiatric Service Dogs for Military-Related PTSD and Associated Symptoms. Am J Orthopsychiatry. 2017;87(4):425–33. doi: 10.1037/ort0000254 28287780

[17] Beetz AM. Theories and possible processes of action in animal assisted interventions. Appl Dev Sci. 2017 Apr;21(2):139–49.

[18] Bulsara M, Wood L, Giles-Corti B, Bosch D. More Than a Furry Companion: The Ripple Effect of Companion Animals on Neighborhood Interactions and Sense of Community. Soc Anim. 2007;15(1):43–56.

[19] Polheber JP, Matchock RL. The presence of a dog attenuates cortisol and heart rate in the Trier Social Stress Test compared to human friends. J Behav Med. 2014 Oct;37(5):860–7. doi: 10.1007/s10865-013-9546-1 24170391

[20] Beck AM. The biology of the human–animal bond. Anim Front. 2014 Jul;4(3):32–6.

[21] Wilson EO. Biophilia [Internet]. Harvard University Press; 1984 [cited 2020 Nov 4]. Available from: https://www.hup.harvard.edu/catalog.php?isbn=9780674074422

[22] Stumbo SP, Yarborough BJH. Preliminary evidence is promising, but challenges remain in providing service dogs to veterans: Commentary on preliminary efficacy of service dogs as a complementary treatment for posttraumatic stress disorder in military members and veterans (O’Haire & Rodriguez, 2018). J Consult Clin Psychol. 2019 Jan;87(1):118–21. doi: 10.1037/ccp0000352 30570307

[23] Imai K, Yamamoto T. Experimental designs for identifying causal mechanisms. J R Stat Soc Ser A Stat Soc. 2013;176(1):5–51.

[24] Kangaslampi S, Peltonen K. Mechanisms of change in psychological interventions for posttraumatic stress symptoms: A systematic review with recommendations. Curr Psychol [Internet]. 2019 Dec 10 [cited 2020 Dec 4]; Available from: http://link.springer.com/10.1007/s12144-019-00478-5

[25] Carr DC, Taylor MG, Gee NR, Sachs-Ericsson NJ. Typologies of older adult companion animal owners and non-owners: moving beyond the dichotomy. Aging Ment Health. 2019 Nov 2;23(11):1452–66. doi: 10.1080/13607863.2018.1503999 30380913

[26] Barcelos AM, Kargas N, Maltby J, Hall S, Mills DS. A framework for understanding how activities associated with dog ownership relate to human well-being. Sci Rep. 2020 Dec;10(1):11363. doi: 10.1038/s41598-020-68446-9 32647301 PMC7347561

[27] Weathers FW, Bovin MJ, Lee DJ, Sloan DM, Schnurr PP, Kaloupek DG, et al. The Clinician-Administered PTSD Scale for DSM–5 (CAPS-5): Development and initial psychometric evaluation in military veterans. Psychol Assess. 2018 Mar;30(3):383–95. doi: 10.1037/pas0000486 28493729 PMC5805662

[28] Ehrhart MG, Ehrhart KH, Roesch SC, Chung-Herrera BG, Nadler K, Bradshaw K. Testing the latent factor structure and construct validity of the Ten-Item Personality Inventory. Personal Individ Differ. 2009;47(8):900–5.

[29] Blevins CA, Weathers FW, Davis MT, Witte TK, Domino JL. The Posttraumatic Stress Disorder Checklist for *DSM-5* (PCL-5): Development and Initial Psychometric Evaluation: Posttraumatic Stress Disorder Checklist for *DSM-5*. J Trauma Stress. 2015 Dec;28(6):489–98. doi: 10.1002/jts.22059 26606250

[30] Bovin MJ, Marx BP, Weathers FW, Gallagher MW, Rodriguez P, Schnurr PP, et al. Psychometric properties of the PTSD Checklist for Diagnostic and Statistical Manual of Mental Disorders–Fifth Edition (PCL-5) in veterans. Psychol Assess. 2016 Nov;28(11):1379–91. doi: 10.1037/pas0000254 26653052

[31] Moshier SJ, Bovin MJ, Gay NG, Wisco BE, Mitchell KS, Lee DJ, et al. Examination of Posttraumatic Stress Disorder Symptom Networks Using Clinician-Rated and Patient-Rated Data. J Abnorm Psychol. 2018 Aug;127(6):541–7. doi: 10.1037/abn0000368 30102064 PMC7059999

[32] Cella D, Riley W, Stone A, Rothrock N, Reeve B, Yount S, et al. The Patient-Reported Outcomes Measurement Information System (PROMIS) developed and tested its first wave of adult self-reported health outcome item banks: 2005–2008. J Clin Epidemiol. 2010 Nov 1;63(11):1179–94. doi: 10.1016/j.jclinepi.2010.04.011 20685078 PMC2965562

[33] Schalet BD, Pilkonis PA, Yu L, Dodds N, Johnston KL, Yount S, et al. Clinical validity of PROMIS Depression, Anxiety, and Anger across diverse clinical samples. J Clin Epidemiol. 2016 May 1;73:119–27. doi: 10.1016/j.jclinepi.2015.08.036 26931289 PMC4928679

[34] Dwyer F, Bennett PC, Coleman GJ. Development of the Monash Dog Owner Relationship Scale (MDORS). Anthrozoös. 2006 Sep 1;19(3):243–56.

[35] Aron A, Aron EN, Smollan D. Inclusion of Other in the Self Scale and the structure of interpersonal closeness. J Pers Soc Psychol. 1992;63(4):596–612.

[36] LaFollette MR, Rodriguez KE, Ogata N, O’Haire ME. Military Veterans and Their PTSD Service Dogs: Associations Between Training Methods, PTSD Severity, Dog Behavior, and the Human-Animal Bond. Front Vet Sci. 2019 Feb 11;6:23. doi: 10.3389/fvets.2019.00023 30805353 PMC6378910

[37] Johnson TP, Garrity TF, Stallones L. Psychometric Evaluation of the Lexington Attachment to Pets Scale (Laps). Anthrozoös. 1992 Sep 1;5(3):160–75.

[38] Blackwell EJ, Bolster C, Richards G, Loftus BA, Casey RA. The use of electronic collars for training domestic dogs: estimated prevalence, reasons and risk factors for use, and owner perceived success as compared to other training methods. BMC Vet Res. 2012;8(1):93. 22748195 10.1186/1746-6148-8-93 PMC3474565

[39] Hiby E, Rooney N, Bradshaw J. Dog training methods: their use, effectiveness and interaction with behaviour and welfare. Anim Welf. 2004;7.

[40] Hsu Y, Serpell JA. Development and validation of a questionnaire for measuring behavior and temperament traits in pet dogs. J Am Vet Med Assoc. 2003 Nov;223(9):1293–300. doi: 10.2460/javma.2003.223.1293 14621216

[41] Robbins ML, Kubiak T. Ecological momentary assessment in behavioral medicine: Research and practice. In: The handbook of behavioral medicine, Vols 1–2. Wiley-Blackwell; 2014. p. 429–46. (Wiley Blackwell handbooks of behavioral neuroscience).

[42] Friard O, Gamba M. BORIS: a free, versatile open-source event-logging software for video/audio coding and live observations. Methods Ecol Evol. 2016;7(11):1325–30.

[43] Zou H, Hastie T. Regularization and variable selection via the elastic net. J R Stat Soc Ser B Stat Methodol. 2005;67(2):301–20.

[44] Calafiore G, El Ghaoui L. Robust maximum likelihood estimation in the linear model. Automatica. 2001 Apr;37(4):573–80.

[45] Long JS, Ervin LH. Using Heteroscedasticity Consistent Standard Errors in the Linear Regression Model. Am Stat. 2000 Aug 1;54(3):217–24.

[46] Kuhn M, Wing J, Weston S, Williams A, Keefer C, Engelhardt A, et al. caret: Classification and Regression Training [Internet]. 2021 [cited 2021 Aug 13]. Available from: https://CRAN.R-project.org/package=caret

[47] Friedman J, Hastie T, Tibshirani R. Regularization Paths for Generalized Linear Models via Coordinate Descent. J Stat Softw [Internet]. 2010 [cited 2021 Aug 13];33(1). Available from: http://www.jstatsoft.org/v33/i01/ 20808728 PMC2929880

[48] Lang DT. RCurl: General Network (HTTP/FTP/. . .) Client Interface for R [Internet]. 2021 [cited 2021 Aug 13]. Available from: https://CRAN.R-project.org/package=RCurl

[49] Ross PT, Hart-Johnson T, Santen SA, Zaidi NLB. Considerations for using race and ethnicity as quantitative variables in medical education research. Perspect Med Educ. 2020 Oct;9(5):318–23. doi: 10.1007/s40037-020-00602-3 32789666 PMC7550522

[50] Weathers FW, Litz BT, Keane TM, Palmieri PA, Marx BP, Schnurr PP. PTSD checklist for DSM-5 (PCL-5) [Internet]. National Center for Posttraumatic Stress Disorder; 2013. Available from: www.ptsd.va.gov

[51] American Psychiatric Association, American Psychiatric Association, editors. Diagnostic and statistical manual of mental disorders: DSM-5. 5th ed. Washington, D.C: American Psychiatric Association; 2013. 947 p.

[52] Crowe TK, Sánchez V, Howard A, Western B, Barger S. Veterans transitioning from isolation to integration: a look at veteran/service dog partnerships. Disabil Rehabil. 2018;40(24):2953–61. doi: 10.1080/09638288.2017.1363301 28805082

[53] Yarborough BJH, Stumbo SP, Yarborough MT, Owen-Smith A, Green CA. Benefits and challenges of using service dogs for veterans with posttraumatic stress disorder. Psychiatr Rehabil J. 2018 Jun;41(2):118–24. doi: 10.1037/prj0000294 29698000

[54] Arata S, Momozawa Y, Takeuchi Y, Mori Y. Important Behavioral Traits for Predicting Guide Dog Qualification. J Vet Med Sci. 2010;72(5):539–45. doi: 10.1292/jvms.09-0512 20009419

[55] Batt LS, Batt MS, Baguley JA, McGreevy PD. Factors associated with success in guide dog training. J Vet Behav. 2008 Jul;3(4):143–51.

[56] Bray EE, Levy KM, Kennedy BS, Duffy DL, Serpell JA, MacLean EL. Predictive Models of Assistance Dog Training Outcomes Using the Canine Behavioral Assessment and Research Questionnaire and a Standardized Temperament Evaluation. Front Vet Sci. 2019 Feb 27;6:49. doi: 10.3389/fvets.2019.00049 30873418 PMC6400848

[57] Höglin A, Van Poucke E, Katajamaa R, Jensen P, Theodorsson E, Roth LSV. Long-term stress in dogs is related to the human–dog relationship and personality traits. Sci Rep. 2021 Dec;11(1):8612. doi: 10.1038/s41598-021-88201-y 33883667 PMC8060293

[58] White N, Mills D, Hall S. Attachment Style Is Related to Quality of Life for Assistance Dog Owners. Int J Environ Res Public Health. 2017 Jun 19;14(6):658. doi: 10.3390/ijerph14060658 28629205 PMC5486344

[59] López-Zerón G, Blow A. The role of relationships and families in healing from trauma. J Fam Ther. 2017;39(4):580–97.

[60] Handlin L, Nilsson A, Ejdebäck M, Hydbring-Sandberg E, Uvnäs-Moberg K. Associations between the Psychological Characteristics of the Human–Dog Relationship and Oxytocin and Cortisol Levels. Anthrozoös. 2012 Jun;25(2):215–28.

[61] MacLean EL, Gesquiere LR, Gee NR, Levy K, Martin WL, Carter CS. Effects of Affiliative Human–Animal Interaction on Dog Salivary and Plasma Oxytocin and Vasopressin. Front Psychol. 2017 Sep 20;8:1606. doi: 10.3389/fpsyg.2017.01606 28979224 PMC5611686

[62] Lue TW, Pantenburg DP, Crawford PM. Impact of the owner-pet and client-veterinarian bond on the care that pets receive. J Am Vet Med Assoc. 2008 Feb 15;232(4):531–40. doi: 10.2460/javma.232.4.531 18279086

[63] MacLean EL, Fine A, Herzog H, Strauss E, Cobb ML. The New Era of Canine Science: Reshaping Our Relationships With Dogs. Front Vet Sci [Internet]. 2021 [cited 2022 Mar 18];8. Available from: https://www.frontiersin.org/article/10.3389/fvets.2021.675782 34336972 10.3389/fvets.2021.675782 PMC8319998

[64] Cobb ML, Otto CM, Fine AH. The Animal Welfare Science of Working Dogs: Current Perspectives on Recent Advances and Future Directions. Front Vet Sci [Internet]. 2021 [cited 2022 Mar 16];8. Available from: https://www.frontiersin.org/article/10.3389/fvets.2021.666898 34722690 10.3389/fvets.2021.666898 PMC8555628

[65] Ziv G. The effects of using aversive training methods in dogs—A review. J Vet Behav. 2017 May 1;19:50–60.

[66] Scotland-Coogan D, Whitworth JD, Wharton T. Outcomes of participation in a service dog training program for veterans with PTSD. Soc Anim. 2020;1(aop):1–22. 10.1080/00981389.2019.1580238 30875483

[67] Williamson L, Dell CA, Osgood N, Chalmers D, Lohnes C, Carleton N, et al. Examining Changes in Posttraumatic Stress Disorder Symptoms and Substance Use Among a Sample of Canadian Veterans Working with Service Dogs: An Exploratory Patient- Oriented Longitudinal Study. J Veterans Stud. 2021 Feb 8;7(1):1.

[68] Shiffman S, Stone AA, Hufford MR. Ecological momentary assessment. Annu Rev Clin Psychol. 2008;4:1–32. doi: 10.1146/annurev.clinpsy.3.022806.091415 18509902

[69] Payne E, Bennett P, McGreevy P. Current perspectives on attachment and bonding in the dog&ndash; human dyad. Psychol Res Behav Manag. 2015 Feb;71. doi: 10.2147/PRBM.S74972 25750549 PMC4348122

[70] Vespa JE. Those Who Served: America’s Veterans From World War II to the War on Terror.: 18.

[71] Smith Y. Pets and Human Diversity: Toward Culturally Competent, Culturally Humble Psychotherapy. In: Clinician’s Guide to Treating Companion Animal Issues [Internet]. Elsevier; 2019 [cited 2021 Jul 28]. p. 477–96. Available from: https://linkinghub.elsevier.com/retrieve/pii/B9780128129623000253

[72] Gray PB, Young SM. Human–Pet Dynamics in Cross-Cultural Perspective. Anthrozoös. 2011 Mar;24(1):17–30.

[73] McClendon J, Dean KE, Galovski T. Addressing Diversity in PTSD Treatment: Disparities in Treatment Engagement and Outcome Among Patients of Color. Curr Treat Options Psychiatry. 2020 Sep;7(3):275–90.

[74] Spoont MR, Sayer NA, Kehle-Forbes SM, Meis LA, Nelson DB. A Prospective Study of Racial and Ethnic Variation in VA Psychotherapy Services for PTSD. Psychiatr Serv. 2017 Mar;68(3):231–7. doi: 10.1176/appi.ps.201600086 27799020

